# Diet-Induced Changes in Functional Disability among People with Multiple Sclerosis: A Secondary Pooled Analysis of Two Randomized Controlled Pilot Trials ^†^

**DOI:** 10.3390/sclerosis2030011

**Published:** 2024-07-04

**Authors:** Allison R. Groux, Elizabeth S. Walker, Farnoosh Shemirani, Jennifer E. Lee, Amanda K. Irish, Linda M. Rubenstein, Linda G. Snetselaar, Warren G. Darling, Terry L. Wahls, Tyler J. Titcomb

**Affiliations:** 1 Department of Internal Medicine, University of Iowa, Iowa City, IA 52340, USA; 2 Department of Biology, University of Iowa, Iowa City, IA 52340, USA; 3 College of Nursing, University of Iowa, Iowa City, IA 52340, USA; 4 Department of Emergency Medicine, University of Iowa, Iowa City, IA 52340, USA; 5 Department of Epidemiology, University of Iowa, Iowa City, IA 52340, USA; 6 Department of Health and Human Physiology, University of Iowa, Iowa City, IA 52340, USA

**Keywords:** multiple sclerosis, modified Paleolithic diet, functional disability, multiple sclerosis functional composite, nine-hole peg test

## Abstract

Emerging evidence links dietary interventions to favorable multiple sclerosis (MS) outcomes; however, evidence for the efficacy of dietary interventions on functional disability remains sparse. Data from two 12-week, randomized, controlled pilot trials were pooled to investigate the efficacy of a modified Paleolithic diet (Paleo) on functional disability, as assessed by the MS Functional Composite (MSFC), among people diagnosed with MS. Pooled baseline-referenced MSFC scores were calculated from the nine-hole peg test (NHPT), timed 25-foot walk (T25FW), and Paced Auditory Serial Addition Test (PASAT) Z-scores. There was no significant difference in the mean change in MSFC scores between groups (*p* = 0.07). In the Paleo group, a significant increase was observed in the MSFC scores (*p* = 0.03), NHPT (*p* < 0.001), and PASAT (*p* = 0.04) Z-scores at 12 weeks, indicating reduced functional disability compared to baseline values. No significant changes were observed within the Control group. Study-specific differences in the MSFC changes between groups were observed. Functional disability was reduced compared to the baseline in the Paleo group, possibly depending on MS type. These results provide preliminary observations on the efficacy of a modified Paleolithic diet for reducing or maintaining functional disability in MS.

## 1. Introduction

Multiple sclerosis (MS) is a debilitating neurodegenerative disease with a complex etiology that affects nearly one million people in the United States [1]. People living with MS report interest in alternative therapies, such as diet, to manage their symptoms and improve health and wellness [2,3]. Surveys have observed that up to half of people with MS report adopting dietary modifications [4,5]. Currently, dietary changes are rarely recommended as standard MS care [6] due, in part, to a lack of evidence for the efficacy of diet in improving disease progression and activity [7,8].

Emerging evidence has linked several dietary interventions to favorable patient-reported outcomes among people with MS. A recent network meta-analysis found that dietary interventions, including the modified Paleolithic diet, recommending a high intake of fruits and vegetables and a reduced intake of ultra-processed foods, reduced fatigue and improved mental and physical quality of life [9]. In addition, several observational studies link adherence to other diets with recommendations for an increased intake of fruits and vegetables and a reduced intake of ultra-processed foods to reduce disability [10–13].

However, another meta-analysis investigating the efficacy of dietary intervention on disability status observed no relationship between dietary intervention and the Kurtzke Expanded Disability Status Scale (EDSS), with a standardized mean difference (with a 95% confidence interval) of −0.19 (−0.40, 0.03) compared to the control [14]. Given this null finding for the effect of dietary interventions on the EDSS, more research is needed to fully elucidate the impact of dietary interventions on disability status among people living with MS. We recently conducted a secondary analysis of a randomized parallel-arm dietary intervention trial investigating the effect of modified Paleolithic and low-saturated-fat diets on fatigue [15], which showed that diet-induced reductions in fatigue mediate improvements in motor dexterity, as assessed by the nine-hole peg test (NHPT), and functional disability, as assessed by the MS Functional Composite (MSFC) [16]. However, this trial did not include a control group and could not be used to establish the efficacy of the modified Paleolithic and low-saturated-fat dietary interventions in reducing functional disability.

Therefore, the objective of the present pooled secondary analysis of two randomized, controlled, pilot trials [17,18] was to preliminarily assess the efficacy of a modified Paleolithic diet in reducing functional disability in MS.

## 2. Materials and Methods

### 2.1. Study 1

#### 2.1.1. Overview

**Figure 1.**
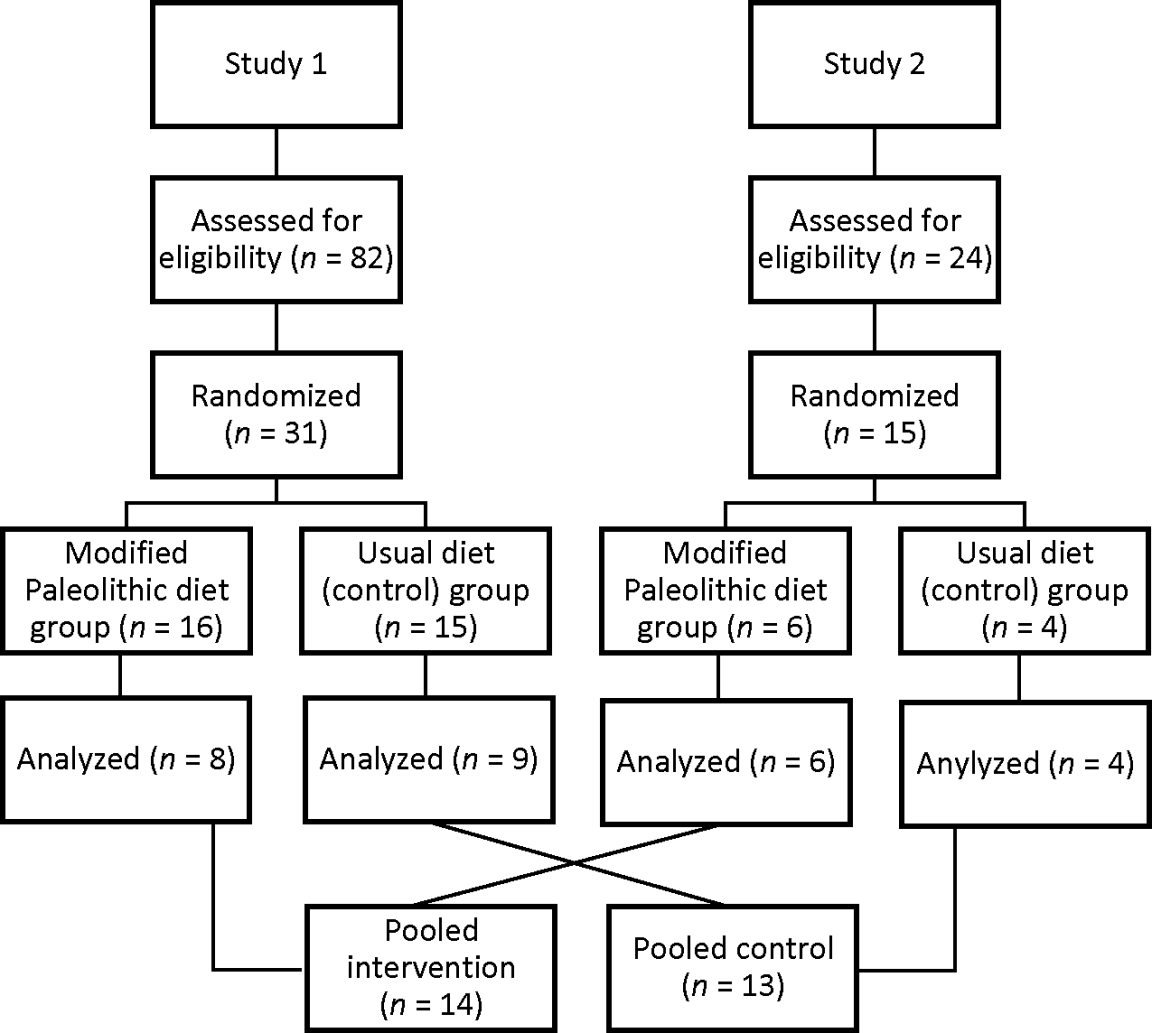
Pooled analysis flowchart. A total of 106 individuals were assessed for eligibility in the two studies combined. Of these, 46 were randomized (22 into a modified Paleolithic diet and 19 into Control). A total of 14 Paleo diet participants and 13 usual diet participants were analyzed.

Study 1 utilized a randomized, controlled pilot trial design to investigate the effect of a modified Paleolithic dietary intervention on the patient-reported and clinical outcomes among people with relapsing–remitting MS (RRMS) [17]. The recruitment of participants included the mass email system at the University of Iowa (UI), the National Multiple Sclerosis Society’s local databases, posters and flyers distributed to local neurology clinics, and word of mouth. This trial was approved by the UI and Iowa City Veteran’s Affairs Medical Center Institutional Review Boards, and written informed consent was obtained from all participants (ClinicalTrials.gov registration: NCT02687919). The participants were randomized based on their fatigue severity scale (FSS) scores into the usual care (Control) or modified Paleolithic diet (Paleo) groups. The study included assessments at baseline and a 12-week follow-up. All 17 participants (8 Paleo and 9 Control) who completed the trial and adhered to the intervention are included in the present secondary analysis (Figure 1).

#### 2.1.2. Participants

The eligibility criteria included an age between 18 and 45 years, stable neurologist-verified RRMS as determined by the 2001 McDonald criteria [19] without medication changes in the 3 months prior to enrollment, and the ability to walk 25 feet with or without the support of an assistive device.

The exclusion criteria included participant self-report of any of the following: cancer, liver disease, kidney disease, diabetes, active heart disease, heart block or arrhythmias, bleeding disorders, concurrent diuretic use, anticoagulant or antiplatelet use, or psychosis or other psychiatric disorders that would potentially impact their ability to perform the study procedures. Participants were additionally excluded for failure to obtain neurologist verification of RRMS or incomplete questionnaires prior to randomization. All the participants were instructed to continue their current MS therapies and medications as instructed by their healthcare providers.

#### 2.1.3. Intervention

The participants in the Paleo group received one follow-up phone call per week for the first 3 weeks of the intervention, followed by calls every other week for the remainder of the study, to aid in diet adherence and ensure the accuracy of the food records. Additionally, participants were permitted to contact the study team for further instruction or assistance.

### 2.2. Study 2

#### 2.2.1. Overview

Study 2 utilized a randomized, waitlist-control trial design to investigate the effect of modified Paleolithic and ketogenic diets on the patient-reported and clinical outcomes in participants with progressive MS or RRMS with significant disability defined by an EDSS score ≥ 4.5 [18]. Participants were recruited by physicians at the Iowa City Veteran’s Affairs Medical Center and the UI Department of Neurology. Potentially eligible patients at the UI Department of Neurology were also mailed a study recruitment letter. Other participants became aware of the study by word of mouth and contacted the study team to participate. The trial was approved by the UI Institutional Review Board, and written informed consent was obtained from all the participants (ClinicalTrials.gov registration: NCT01915433). Participants were randomized 1:1:1 into 3 arms: (1) a modified Paleolithic diet (Paleo); (2) a ketogenic diet; or (3) usual diet/waitlist-control (Control). Study assessments were conducted at baseline and 12 weeks. The ketogenic diet group was excluded from the present analysis given the lack of this group in Study 1. The remaining 10 participants (6 Paleo and 4 Control) who completed the trial are included in the present secondary pooled analysis (Figure 1).

#### 2.2.2. Participants

The eligibility criteria included an age between 30 and 65 years, a formal MS diagnosis based on the 2010 McDonald criteria [20] of primary progressive MS, secondary progressive MS, or RRMS with an EDSS score ≥ 4.5, the ability to walk 25 feet in less than 60 s, and severe fatigue (defined as Modified Fatigue Impact Scale score ≥ 38).

The exclusion criteria included clinically significant liver, kidney, or heart disease, anti-platelet or anticoagulant therapy, severe psychiatric disorders, diabetes (current or prior), the inability to record daily weights at home, any change in prescription medication in the 3 months leading up to enrollment, adherence to a vegetarian or Paleolithic diet (elimination of all grains, dairy, and legumes) prior to the study enrollment, the inability to afford a possible 30% increase in grocery bill expenses, alanine amino transferase greater than 2 times the normal level (or elevated creatine), the inability to give informed consent, a body mass index (BMI) of less than 19, unwillingness to follow the study diet protocol, or chronic diarrhea.

#### 2.2.3. Intervention

After randomization, a focused medical history was obtained from all the participants. Registered dietitian nutritionists (RDNs) then provided education on a modified Paleolithic diet. The RDNs made nutrition counseling calls 2–3 days after the baseline visit and weekly thereafter for the subsequent three weeks. After the participants in the intervention arms were educated by the RDNs, they were provided the option to contact the RDNs throughout the study with diet-related questions. The intervention arm participants were also given diet-specific supplies and information.

### 2.3. Modified Paleolithic Diet

A modified Paleolithic diet recommends 6–9 daily servings of fruits and vegetables comprising 2–3 servings of each of the following categories: green leafy vegetables, sulfur-rich vegetables, and intensely colored fruits or vegetables [21]. Organ meat, omega-3-rich fish, seaweed, and algae (e.g., spirulina, chlorella, Klamath blue green) are also encouraged. This diet eliminates the consumption of dairy, eggs, and gluten-containing grains (e.g., wheat, barley, rye). In Study 1, the participants were also instructed to eliminate potatoes and legumes (e.g., beans, lentils, peanuts, soy; [17]).

### 2.4. Outcomes

The MS Functional Composite (MSFC) is an objective measure of physical and cognitive function among people living with MS [22]. The MSFC was calculated as the mean of the NHPT, T25FW, and PASAT baseline-referenced Z-scores. Increased MSFC scores at follow-up indicate reduced functional disability. In both studies, the components of the MSFC were assessed at baseline and again at 12 weeks. Clinically meaningful changes were defined as a ≥0.5-point increase for the MSFC score [23], ≥20% reductions in time for the NHPT and T25FW [24], or an increase of one standard deviation (SD) for the PASAT [25]. Fatigue was assessed using the Fatigue Severity Scale (FSS).

### 2.5. Statistical Analysis

Descriptive statistics were calculated for every variable at enrollment using frequencies, means ± standard error (SE), and medians (interquartile range). The distributions of continuous variables were evaluated for normality by graphical observation, and outliers were checked for accuracy.

Generalized linear mixed models [26] were used to estimate and test the interacting effects of diet and time on the outcome measures while accounting for repeated measures for each participant using a random effect. The models for the outcomes with normal distributions specified the identity functions. Point estimates, 95% confidence intervals, and *p*-values of the group-specific mean changes in the outcome measures over visits were generated for each model. For the primary MSFC analysis, pooled data from all the participants completing 12-week assessments were included. Models for the analysis of the pooled data were adjusted for each study.

Additional analyses were conducted for each of the MSFC individual components, the NHPT, T25FW, and PASAT Z-scores. Sensitivity analyses were also conducted for each trial independently. The proportion of participants in each group exceeding the thresholds for clinically meaningful changes was compared using Fisher’s exact test. The relationship between fatigue and functional outcomes was explored with causal mediation analyses [27].

All the analyses were conducted with two-sided tests (α = 0.05) using SAS software (version 9.4, SAS Institute, Inc.; Cary, NC, USA).

## 3. Results

The mean age of the participants in Study 1 was 35.4 ± 5.7 years for the Paleo group and 37.1 ± 3.7 years for the Control group (Table 1). Most of the participants in both groups were female (87.5% Paleo, 88.9% Control). In Study 1, the mean MSFC scores and the PASAT Z-scores at baseline were lower in the Paleo group compared to the Control (*p* = 0.04 and *p* < 0.001, respectively). The participants in Study 2 had a mean age of 50.3 ± 9.5 years and 54.5 ± 11.8 years for the Paleo and Control groups, respectively. Half of the participants (50.0%) in the Control group and 33.3% in the Paleo group were female.

For the pooled sample, no differences were observed between groups for the MSFC or its components. The mean 12-week change in mean MSFC scores was not different between groups (0.27 ± 0.15; *p* = 0.07; Table 2). Among the Paleo group, the mean MSFC scores increased from −0.32 ± 0.20 at baseline to −0.06 ± 0.20 at 12 weeks (*p* = 0.03). There was no change in the mean MSFC scores within the Control group. For the change in mean NHPT Z-scores, the Paleo group demonstrated a greater 12-week change compared with the Control group (0.35 ± 0.14; *p* = 0.02). In the Paleo group, the mean NHPT Z-scores increased from −0.42 ± 0.17 at baseline to 0.03 ± 0.17 at 12 weeks (*p* < 0.001). No change was observed in the mean NHPT Z-scores within the Control group. The mean PASAT Z-scores increased in the Paleo group from −0.19 ± 0.30 at baseline to 0.19 ± 0.31 at 12 weeks (*p* = 0.04). No changes were observed within the Control group and no differences were observed between groups in terms of the PASAT Z-scores. No differences were observed in the mean T25FW Z-scores within or between groups. The participants in the Paleo group were more likely to experience a clinically meaningful improvement in the MSFC compared to the Control group (*p* = 0.04; Table 3), but there were no differences in the proportion with a clinically meaningful change in the NHPT, T25FW, or PASAT. Of the participants with clinically meaningful reductions in functional disability, 80% were enrolled in Study 1, and 100% of the participants with clinically meaningful increases in functional disability were enrolled in Study 2.

In an analysis restricted to only Study 1, the 12-week change in the mean MSFC score was higher in the Paleo group compared with the Control group (0.65 ± 0.23; *p* = 0.004); however, the baseline MSFC values differed between groups (0.66 ± 0.32; *p* = 0.04; Table 2). The mean MSFC scores increased from −0.35 ± 0.27 at baseline to 0.56 ± 0.18 at 12 weeks in the Paleo group (*p* < 0.001). In addition, the mean MSFC scores increased from 0.31 ± 0.16 at baseline to 0.58 ± 0.16 at 12 weeks in the Control group (*p* = 0.05). The mean NHPT Z-scores increased from −0.34 ± 0.37 at baseline to 0.72 ± 0.34 at 12 weeks in the Paleo group (*p* < 0.001) and from 0.30 ± 0.26 at baseline to 0.60 ± 0.20 at 12 weeks in the Control group (*p* = 0.005); however, the 12-week mean change was higher in the Paleo group compared with the Control group (0.75 ± 0.22; *p* < 0.001). In the Paleo group, the mean PASAT Z-scores increased from −0.70 ± 0.24 at baseline to 0.07 ± 0.37 at 12 weeks (*p* = 0.01), and no change was observed within the Control group. The mean PASAT Z-score values differed between groups at baseline (*p* < 0.001) and 12 weeks (*p* = 0.05), but there was no difference in the mean change between groups. In the Paleo group, the mean T25FW Z-scores increased from −0.02 ± 0.39 at baseline to 0.90 ± 0.29 at 12 weeks (*p* = 0.002); however, no change was observed within the Control group, and there were no differences observed between groups.

In an analysis restricted to only Study 2, the mean change in MSFC scores did not differ between groups; however, the values significantly decreased from 0.15 ± 0.27 at baseline to −0.14 ± 0.24 at 12 weeks in the Control group (*p* = 0.02; Table 2). No change was observed in the mean MSFC scores among the Paleo group. No changes were observed in the mean NHPT Z-scores within the Paleo or Control groups; however, the 12-week mean change was 0.37 ± 0.19 higher in the Paleo group compared to the Control group (*p* = 0.05). There were no differences observed in the mean T25FW or PASAT Z-scores within or between groups. There were no differences in the baseline scores between the groups for any of the MSFC components.

There were no significant mediation effects of fatigue on the outcomes in the pooled sample.

## 4. Discussion

In this secondary pooled analysis of two randomized, controlled pilot trials, the 12-week mean change in MSFC scores was not different between groups; however, the mean NHPT Z-scores increased more in the Paleo group compared with the Control group. In Study 1 [17], there was a greater increase in the mean MSFC scores and NHPT Z-scores in the Paleo group compared with the Control group. In Study 2 [18], there was a higher increase only in the mean NHPT Z-scores in the Paleo group compared with the Control group.

The present findings are corroborated by a previous randomized, parallel-arm trial comparing modified Paleolithic elimination and low-saturated-fat diets, which showed significant within-group increases in the mean NHPT Z-scores among participants with RRMS at 24 weeks [16]. The previous trial also observed within-group increases in the mean MSFC scores, which is notable given that in the present study, the mean MSFC scores increased only in Study 1, which was also conducted among participants with RRMS. This observation suggests that a diet-induced improvement in functional disability may be dependent on the MS type or possibly the underlying severity of the disease or age of the participant. This idea is further supported by the results of Study 2, which demonstrated worsening functional disability in the Control group, whereas no change was observed in the Paleo group among a sample of older participants with progressive MS or RRMS with moderate to severe disability (EDSS ≥ 4.5). The observation that the Paleo group maintained their mean MSFC scores in Study 2 is noteworthy, as a continued long-term decline in functional disability would be expected in people living with progressive MS [28]; however, these findings need to be replicated given the short duration of the studies included in this secondary analysis.

Other diets that recommend a high intake of fruits and vegetables and a reduced intake of ultra-processed foods have also been shown to impact functional disability in MS. Notably, a 12-month randomized controlled trial observed within-group increases in the mean MSFC scores following a low-fat plant-based diet intervention; however, the improvement in mean MSFC scores did not differ from the control, possibly due to the inclusion of an exercise intervention in both groups [29]. In addition, a cross-sectional analysis observed that adherence to a Mediterranean diet is linked to higher MSFC scores [12]. These diets, as well as a modified Paleolithic diet, were also shown to reduce fatigue and improve physical and mental quality of life among people living with MS in a recent systematic review and network meta-analysis [9]. Given these promising findings regarding the impact of diet on MS outcomes and the immense interest in diet among people living with MS [2], experts in the field have called for the inclusion of RDNs in the care of people living with MS [30,31].

The strengths of this secondary pooled analysis include the use of an objective measure of functional disability, controlled trial designs, the inclusion of multiple types of MS, and robust analytical methods. However, this secondary pooled analysis of two pilot trials has several limitations, including the small sample sizes, the short duration of the dietary intervention, the slightly differing definitions of a modified Paleolithic diet, the differing baseline ages and functional disability scores of the participants between trials, and the wide range of exclusion criteria in each trial. The possibility of random effects in view of the sample size limits any substantive inference on the results of the study. Notably, the null finding of fatigue-mediated changes in functional disability was presumably driven by a lack of power. Additionally, other important variables such as the EDSS scores, DMT use, the brain lesion load, disease duration, BMI, comorbidity burden, dietary intake, and other lifestyle variables such as smoking status were not consistently collected across trials, and as such, they are not able to be accounted for in the present analysis. Furthermore, the pooled data from two pilot studies with small sample sizes lead to an increased risk of incorrectly concluding significance due to type one errors, which necessitates caution in interpreting these results. However, the findings in the present study are supported by those from our previous trial [16] and therefore warrant further investigation. The two pilot trials also utilized two different versions of the McDonald criteria which have since been updated; therefore, cases of an incorrect diagnosis cannot be ruled out in the study samples. Finally, one of the authors has a conflict of interest regarding the modified Paleolithic diet (see “Conflicts of Interest” statement).

## 5. Conclusions

The present results provide preliminary observations on the efficacy of a modified Paleolithic dietary intervention for reducing functional disability in MS. The observation in the Paleo groups that functional disability was reduced in Study 1 in the participants with RRMS and was maintained in Study 2 in older participants with progressive MS and greater functional disability suggests that implementing dietary interventions earlier in the disease course may maximize the benefits of the dietary intervention. Future well-designed randomized controlled trials with larger sample sizes are needed to confirm these findings.

## Figures and Tables

**Table 1.** Baseline characteristics.^1,2^

	Study 1		Study 2	
Characteristic	Control	Paleo	Control	Paleo

Sex (female)	8 (88.9)	7 (87.5)	2 (50.0)	2 (33.3)
Age (years)	37.1 ± 3.7	35.4 ± 5.7	54.5 ± 11.8	50.3 ± 9.5
MS type				
-Relapsing	9 (100)	8 (100)	1 (25)	1 (16.7)
-Progressive	0	0	3 (75)	5 (83.3)
T25FW (seconds)	4.7 ± 0.5	6.1 ± 2.4	12.6 ± 3.2	21.0 ± 14.4
NHPT (dominant; seconds)	19.6 ± 2.0	21.0 ± 3.9	34.5 ± 18.2	38.4 ± 13.9
NHPT (non-dominant; seconds)	20.4 ± 3.6	24.4 ± 4.5	30.5 ± 6.5	39.5 ± 17.1
PASAT (0 to 60)	53.2 ± 5.0	44.9 ± 4.5	41.3 ± 11.1	49.3 ± 12.5

1 All values shown as mean ± SD or N (%).

2 Abbreviations: MS, multiple sclerosis; NHPT, nine-hole peg-test; PASAT, paced auditory serial addition test; T25FW, timed 25-foot walk.

**Table 2.** Change in multiple sclerosis functional composite scores during 12-week dietary intervention trial.^1,2^

	Control			Paleo			
Pooled	Baseline	12-weeks	Change^3^	Baseline	12-weeks	Change^3^	p-value^4^

MSFC	0.00 ± 0.13	−0.01 ± 0.14	−0.01 ± 0.08	−0.32 ± 0.20	−0.06 ± 0.20	0.26 ± 0.12*	0.07
NHPT	−0.01 ± 0.18	0.09 ± 0.15	0.10 ± 0.07	−0.42 ± 0.17	0.03 ± 0.17	0.45 ± 0.12***	0.02
T25FW	−0.03 ± 0.12	−0.08 ± 0.10	−0.05 ± 0.04	−0.34 ± 0.27	−0.41 ± 0.33	−0.07 ± 0.17	0.92
PASAT	0.04 ± 0.28	−0.03 ± 0.36	−0.07 ± 0.17	−0.19 ± 0.30	0.19 ± 0.31	0.39 ± 0.18*	0.07
							
Study 1							

MSFC	0.31 ± 0.16	0.58 ± 0.16	0.27 ± 0.13*	−0.35 ± 0.27	0.56 ± 0.18	0.91 ± 0.18***	0.005
NHPT	0.30 ± 0.26	0.60 ± 0.20	0.30 ± 0.11**	−0.34 ± 0.37	0.72 ± 0.34	1.06 ± 0.19***	<0.001
T25FW	0.01 ± 0.27	0.25 ± 0.29	0.23 ± 0.21	−0.02 ± 0.39	0.90 ± 0.29	0.91 ± 0.29**	0.06
PASAT	0.62 ± 0.25	0.88 ± 0.18	0.26 ± 0.17	−0.70 ± 0.24	0.07 ± 0.37	0.77 ± 0.30**	0.14
							
Study 2							

MSFC	0.15 ± 0.27	−0.14 ± 0.24	−0.29 ± 0.12*	−0.10 ± 0.33	−0.08 ± 0.37	0.02 ± 0.18	0.16
NHPT	0.43 ± 0.55	0.19 ± 0.46	−0.24 ± 0.13	−0.29 ± 0.28	−0.16 ± 0.33	0.13 ± 0.13	0.05
T25FW	0.43 ± 0.12	0.27 ± 0.10	−0.15 ± 0.09	−0.29 ± 0.46	−0.47 ± 0.56	−0.19 ± 0.32	0.92
PASAT	−0.40 ± 0.40	−0.88 ± 0.50	−0.48 ± 0.25	0.27 ± 0.39	0.39 ± 0.39	0.12 ± 0.22	0.07

1 All values shown as mean ± SEM.

2 Abbreviations: MSFC, multiple sclerosis functional composite; NHPT, nine-hole peg-test; PASAT, paced auditory serial addition test; T25FW, timed 25-foot walk.

3 Within-group significance indicated by

* for p ≤ 0.05,

** for p ≤ 0.01,

*** for p ≤ 0.001.

4 Between-group difference in 12-week change.

**Table 3.** Frequency (%) with clinically meaningful 12-week changes in functional disability.^1^

		Control			Paleo			
	Threshold^2^	Worsened	No Change	Improved	Worsened	No Change	Improved	p-Value^3^

MSFC	0.5	1 (7.69)	12 (92.3)	0	1 (7.14)	8 (57.1)	5 (35.7)	0.04
NHPT	20%	0	13 (100)	0	0	11 (78.6)	3 (21.4)	0.23
T25FW	20%	2 (15.4)	11 (84.6)	0	2 (14.3)	9 (64.3)	3 (21.4)	0.30
PASAT	1 SD	1 (7.69)	12 (92.3)	0	0	11 (78.6)	3 (21.4)	0.23

1 Abbreviations: MSFC, multiple sclerosis functional composite; NHPT, nine-hole peg-test; PASAT, paced auditory serial addition test; T25FW, timed 25-foot walk.

2 Threshold for clinically meaningful change.

3 Determined by Fischer’s Exact test.

## Data Availability

The data presented in this study are available on request from the corresponding author due to the small sample size and the protection of the anonymity of the participants.

## Footnotes

**Institutional Review Board Statement:** The included trials were conducted in accordance with the Declaration of Helsinki and approved by the University of Iowa Institutional Review Board (#201302829 and #201610794).
